# 408. Clinical Spectrum of Pediatric Long COVID and Impact of COVID-19 Vaccines on the Clinical Course

**DOI:** 10.1093/ofid/ofad500.478

**Published:** 2023-11-27

**Authors:** Dina Kamel, Jeffrey Bender, David Warburton, John C Wood, Sindhu Mohandas

**Affiliations:** Children's Hospital Los Angeles, Covina, California; Children's Hospital Los Angeles, Covina, California; Children's Hospital Los Angeles, Covina, California; Children's Hospital Los Angeles, Covina, California; Children Hospital Los Angeles, los angeles, CA

## Abstract

**Background:**

Long COVID is a well-recognized, emerging health problem following COVID-19 infection. It presents with a wide range of ongoing, new, or returning symptoms following COVID-19 infection. Symptoms can last from weeks to months, significantly impacting patients’ quality of life. There is no precise definition for long COVID making it difficult to diagnose. Little is known about long COVID in children and the effect of COVID-19 vaccine on its course. This study aims to describe the most common symptoms in pediatric patients and to study the impact of vaccination on the course of long COVID.

**Methods:**

A retrospective analysis of all children aged 0-21 years seen in the COVID Recovery Clinic at Children’s Hospital of Los Angeles from August 2021 to April 2023. Data were abstracted using a standardized tool and analyzed using SPSS 28.

**Results:**

Overall, 107 patients were identified with long COVID. Of these, 51 (47.7%) were females with a median age of 14.1 years (IQR 11.1-15.9). Data regarding race and ethnicity were limited. The most commonly reported symptoms were fatigue (94.4%), headache (73.6%), exercise intolerance (57%), dizziness (49.5%), brain fog (41.1%), shortness of breath (31.8%), musculoskeletal pain (30.8%), chest pain and palpitations (29%). Symptoms developed immediately after COVID-19 infection in 56 (52.3%) of patients, within 1-4 weeks in 37 (34.7%), and after 4 weeks in 14 (13%) of them. At the time of presentation to the clinic, 57 (53.3%) were vaccinated against COVID-19, and 35 (61.4%) of them received the vaccine after the onset of long COVID symptoms. Of those 35 patients, 51.4% reported no change in symptoms after vaccine administration, 42.9% reported symptom improvement and 5.7% had progression of symptoms.

**Figure:**
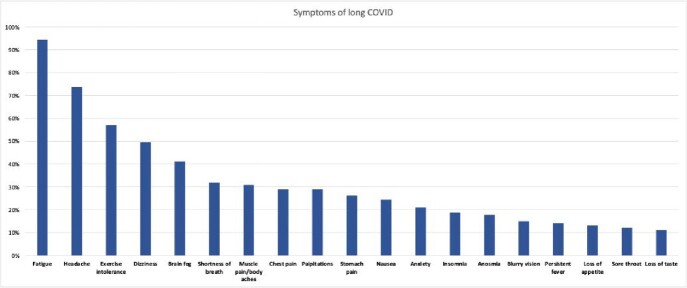

Figure (1)

**Figure:**
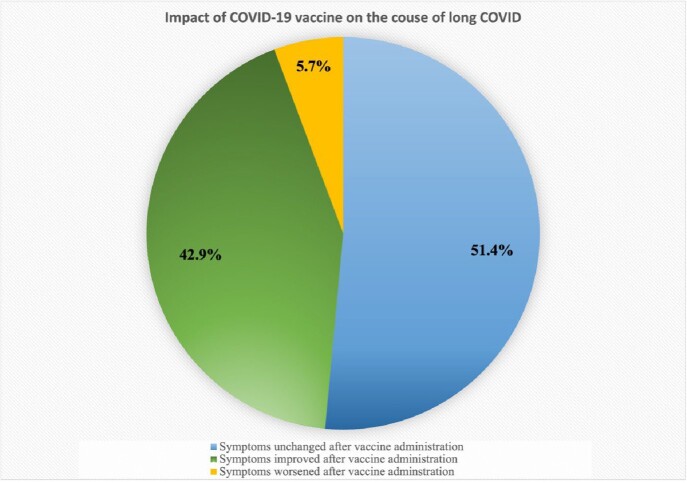

**Figure (2)**

**Conclusion:**

Long COVID is a growing well-recognized problem in children after COVID-19 infection. In our large cohort of pediatric patients, we identified fatigue and headache as the predominant presenting symptoms. COVID-19 vaccine led to symptomatic improvement in a proportion of patients. Longer prospective follow-up is needed to help better understand the impact of long COVID on children. COVID-19 vaccines should be further investigated in a systematic fashion as a possible therapeutic intervention in children with long COVID.

**Disclosures:**

**All Authors**: No reported disclosures

